# Measurement properties of a novel survey to assess stages of organizational readiness for evidence-based interventions in community chronic disease prevention settings

**DOI:** 10.1186/1748-5908-7-65

**Published:** 2012-07-16

**Authors:** Katherine A Stamatakis, Amy McQueen, Carl Filler, Elizabeth Boland, Mariah Dreisinger, Ross C Brownson, Douglas A Luke

**Affiliations:** 1 Division of Public Health Sciences and Alvin J. Siteman Cancer Center, Washington University School of Medicine, St. Louis, MO, USA; 2 Division of Health Behavior Research, Washington University School of Medicine, St. Louis, MO, USA; 3 Prevention Research Center in St. Louis, Brown School, Washington University, St. Louis, MO, USA; 4 School of Medicine, University of Missouri-Columbia, Columbia, MO, USA; 5 Center for Tobacco Policy Research, Brown School, Washington University, St. Louis, MO, USA

**Keywords:** Measurement tool, Chronic disease prevention, Evidence-based practice, Confirmatory factor analysis, Dissemination, Implementation

## Abstract

**Background:**

There is a great deal of variation in the existing capacity of primary prevention programs and policies addressing chronic disease to deliver evidence-based interventions (EBIs). In order to develop and evaluate implementation strategies that are tailored to the appropriate level of capacity, there is a need for an easy-to-administer tool to stage organizational readiness for EBIs.

**Methods:**

Based on theoretical frameworks, including Rogers’ Diffusion of Innovations, we developed a survey instrument to measure four domains representing stages of readiness for EBI: awareness, adoption, implementation, and maintenance. A separate scale representing organizational climate as a potential mediator of readiness for EBIs was also included in the survey. Twenty-three questions comprised the four domains, with four to nine items each, using a seven-point response scale. Representatives from obesity, asthma, diabetes, and tobacco prevention programs serving diverse populations in the United States were surveyed (N = 243); test-retest reliability was assessed with 92 respondents.

**Results:**

Confirmatory factor analysis (CFA) was used to test and refine readiness scales. Test-retest reliability of the readiness scales, as measured by intraclass correlation, ranged from 0.47–0.71. CFA found good fit for the five-item adoption and implementation scales and resulted in revisions of the awareness and maintenance scales. The awareness scale was split into two two-item scales, representing community and agency awareness. The maintenance scale was split into five- and four-item scales, representing infrastructural maintenance and evaluation maintenance, respectively. Internal reliability of scales (Cronbach’s α) ranged from 0.66–0.78. The model for the final revised scales approached good fit, with most factor loadings >0.6 and all >0.4.

**Conclusions:**

The lack of adequate measurement tools hinders progress in dissemination and implementation research. These preliminary results help fill this gap by describing the reliability and measurement properties of a theory-based tool; the short, user-friendly instrument may be useful to researchers and practitioners seeking to assess organizational readiness for EBIs across a variety of chronic disease prevention programs and settings.

## Introduction

In the United States, chronic disease is the most common cause of disability and death, with cancer, heart disease, and cerebrovascular disease alone accounting for over half of all deaths [1] and consuming over 80% of the healthcare budget in the United States [2]. While community-level programs and policies in prevention and control hold great promise for reducing the burden of chronic disease [3-5], community prevention programs are increasingly pressured to stretch staff and budgets across a broad range of activities and responsibilities, emphasizing the need to direct scarce resources to the most effective programs.

Evidence-based interventions (EBIs) in chronic disease prevention are comprised of increasing numbers of programs and policies that have been research-tested and are ready for dissemination to community and public health practice settings (the Guide to Community Preventive Services [a.k.a., the Community Guide], the Guide to Clinical Preventive Services, Cancer Control PLANET, Research-tested Intervention Programs [6-9]). However, there is reason to believe that the best available evidence is not reaching or being integrated into all practice settings. In a survey of state and local public health practitioners, only 30% of practitioners at the local level had heard of the Community Guide [10], a standard for recommended EBIs in community health [11]. Among state-level practitioners, almost 90% of whom had heard of the Community Guide, a much smaller proportion reported making changes to existing (20%) or new programs (35%) on the basis of Community Guide recommendations [10]. Thus, these data indicate that the challenge for improving uptake of EBIs extends beyond simply spreading awareness to include integration into practice settings and suggest that readiness for EBIs may vary across settings.

Conceptualizing public health organizational readiness for EBIs requires drawing from a broader understanding of how evidence is used in public health practice as well as processes underlying the movement of research evidence into practice settings. Theoretical frameworks that lay the groundwork for these components of readiness include evidence-based public health [12] and the stages of innovation diffusion [13-17]. Evidence-based public health practice has been described as applying the best available, scientifically rigorous, and peer-reviewed evidence (*i.e.*, EBIs), but it also includes using data and information systems systematically, applying program-planning frameworks, engaging the community in assessment and decision making, conducting sound evaluation, and disseminating what is learned to key stakeholders and decision makers [12]. The conceptualization of stages characterizing the process of uptake and integration of EBIs is grounded in Rogers’ theory of Diffusion of Innovations [14,16] and the application of these tenets in program planning and evaluation, most notably, as exemplified by the RE-AIM framework (*i.e.*, reach, effectiveness, awareness, implementation and maintenance) [15,17]. Together, these frameworks and theoretical models can be applied to the development of constructs for characterizing the stages of a public health organization’s readiness for EBIs, from awareness to adoption, implementation, and maintenance.

One of the central challenges for moving EBIs for chronic disease prevention into community and public health practice settings may be the variability in organizational readiness, but the lack of a tool to measure organizational readiness for EBIs currently hinders the ability to test this hypothesis. The aim of this article is to describe the development, measurement properties, and potential uses of a novel survey instrument used to measure stages of organizational readiness for EBIs, designed to be brief and generalizable across chronic disease prevention program areas.

## Methods

### Study participants

Respondents were sampled to represent program areas in chronic disease prevention, based on the presumption that they could provide an assessment of supports within the larger organization as pertaining to their ability to incorporate EBIs into practice. Since, to our knowledge, there is no existing current database of staff in specific chronic disease program areas in local public health departments and community organizations across the country, we relied on a combination of purposive and snowball sampling to recruit participants. A first set of respondents to the survey were recruited directly through purposive sampling of members of the National Association of Chronic Disease Directors (NACDD), state health department contacts, and their coworkers in the asthma control, diabetes prevention, obesity prevention, and tobacco control fields. After initial contacts, further respondents were selected through snowball sampling [18]. A subset of obesity prevention respondents represented organizations to which the authors are providing technical assistance on dissemination. These 19 organizations were funded through the Missouri Foundation for Health under the Healthy & Active Communities grant to create Model Practice Building (MPB) interventions. The survey (hosted and administered by Qualtrics, Inc.) was distributed by email to all respondents that were known by the researchers. In order to avoid respondents ignoring emails from an unknown sender, those organizations that had not had previous contact with the researchers were first sent a personal email or called over the telephone to identify the relevant staff members to complete the online survey.

The final sample, collected between February and June 2010, included respondents from state health departments, local health departments, and community-based organizations. Of the 393 individuals contacted, 277 took the resulting survey. After removing 34 respondents with incomplete data, the final analytic sample was 243 individuals representing 164 organizations, resulting in a response rate of 62%. A subset of respondents completed the survey a second time one to two months after initial survey administration (n = 92 [65 organizations], response rate 59%) in order to assess test-retest reliability. The study was reviewed and approved by the Washington University in St. Louis Institutional Review Board.

### Measures

The survey instrument was developed based on the underlying staged frameworks articulated in the Diffusion of Innovations theory [16], RE-AIM [17], and a hybrid of these frameworks in Briss *et al.*[19]. This framework was further developed in the current project in the report of the qualitative factors that contribute to or inhibit movement along a staged framework [20].

Specific items for the survey instrument were developed primarily from three sources (which themselves cited Rogers [16] in addition to other above-mentioned frameworks): Steckler and colleagues’ study of the dissemination of tobacco prevention curricula [21], Jacob and colleagues’ study of evidence-based interventions among state-level public health practitioners [22], and the Center for Tobacco Policy Research Program Sustainability Assessment Tool [23]. From these questionnaires and based on input from a group of experts assembled for this study, a set of questions was developed (54 items in initial draft). The questionnaire was further refined based on results of cognitive response testing [24-27] of a group of key stakeholders from funding agencies and practice settings (n = 11). In cognitive response testing, we sought to determine the following: (1) question comprehension (*i.e.*, What does the respondent think the question is asking? What do specific words or phrases in the question mean to the respondent?); (2) information retrieval (*i.e.*, What information does the respondent need to recall from memory in order to answer the question? How do they retrieve this information?); and (3) decision processing (*i.e.*, How do they choose their answer?).

Stages were operationalized based on the items selected during the above-described process of survey development in order to measure stages as latent constructs, with a focus on using as few items as possible to retain user-friendliness of the questionnaire. The resulting four stages were defined as follows: (1) *awareness* as recognition of need and availability of sources for EBIs, which included four items that assessed whether the community considered the health issue to be a problem, its view of solutions, and the extent of awareness of EBIs among agency leadership and staff; (2) *adoption* as decision making based on evidence, which included five items that assessed the extent of using evidence in decision making, support from leadership, and access to technical assistance; (3) *implementation* as carrying out and adapting interventions to meet community needs, which included five items that assessed the extent to which the agency is able to adopt EBIs, having resources and skills needed for implementation, and support from leadership and the community for implementing EBIs; and (4) *maintenance* as the existing embedded activities and resources the organization has to support ongoing EBIs, which included nine items that assessed the extent to which the agency assesses community health needs, conducts evaluation of interventions and disseminates findings, has a network of partners and diverse funding sources, and has policies and procedures to ensure proper allocation of funding. A final contextual domain, “organizational climate” (three items), separate from the readiness scale domains, assessed the ability of the organization—independent from the intervention—to react, change, and adapt to new challenges, needs of the community, and a changing evidence base. A total of 26 questions comprised the four domains and additional contextual domain; all were measured with a seven-point Likert scale. The full survey instrument is available as an appendix (Additional file 1).

### Confirmatory factor analysis

Data were analyzed using a series of confirmatory factor analyses (CFAs) in SPSS AMOS 16.0 (IBM, Armonk, NY). We chose a confirmatory rather than an exploratory approach because we identified items for each stage *a priori* and preferred a more theory-driven test of our model [28,29]. Full-information maximum likelihood (FIML) estimation was used to include all available data. FIML is the recommended estimation method of choice when the data are missing at random and may be less biased than other multivariate approaches when missing data are not ignorable [30-32].

We tested an *a priori* four-factor model, with each stage modeled as a latent factor, but also allowed for improvements and modifications, including alternative factor structures, adding error covariances, and removing poor-performing items (*i.e.*, low factor loading or cross-loading). Correlations between factors also were examined. We used multiple fit indices to evaluate model fit: the chi-square/degrees of freedom, comparative fit index (CFI), and root mean square error of approximation (RMSEA) and its associated 90% confidence interval. CFI values between 0.90–0.95 or above suggest adequate to good fit [33,34] and RMSEA values <0.06 suggest good model fit [33]. Finally, after determination of the final model structure for stages of readiness, we examined the correlations between organizational climate (modeled as a three-item latent factor) and having a university affiliation (modeled as binary exogenous variable) in relation to the factors from the final model.

Additional analyses were performed using STATA version 11 (StataCorp LP, College Station, TX) [35]. Descriptive statistics on characteristics of survey respondents were based on frequency distributions. Test-retest reliability was assessed with the intraclass correlation (ICC) statistic [36]. Cronbach’s alpha was also computed for each of the scales to provide a commonly used metric of internal consistency. Finally, mean scale scores were compared across program types to provide a preliminary assessment of construct validity.

## Results

Descriptive statistics of the respondents and their respective organizations and programs are provided in Table 1.

**Table 1:** Description of surveyed programs and respondents (n = 243)

	**Frequency**	**%**
Organizational characteristics		
Agency/organization type		
State health department	106	43.8
Local health department	52	21.5
Healthcare provider	16	6.6
Community-based organization	32	13.2
Other	36	14.9
Prevention program type		
MPB obesity grantees	31	13.0
Other obesity	45	18.8
Tobacco	92	38.5
Diabetes	42	17.6
Asthma	29	12.1
Geographic region served by intervention		
Urban	34	14.0
Suburban	67	27.7
Rural	11	4.5
Combination urban, suburban, and/or rural	130	53.7
Affiliation with a university		
Yes	82	34.3
No	157	65.7
Respondent characteristics		
Length of time respondent worked with intervention		
<5 years	128	52.9
5–10 years	69	28.5
11–15 years	20	8.3
>15 years	25	10.3
Respondent’s position in agency		
Program manager	166	68.6
Direct service staff	43	17.8
Program support and evaluation	29	12.0
Academic researcher	2	0.8
Academic educator	2	0.8

MPB = Model Practice Building.

### Measurement model development

The measurement model was first tested based on the initial hypothesized correlated four-factor structure representing the four stages of organizational readiness. As Table 2 shows, the initial model had poor fit across all fit indices. In model 1, most modification indices were relevant to the awareness and maintenance scale items, which caused us to recognize the “natural” split for the items (*i.e.*, community and agency subscales of awareness, resource and evaluation subscales of maintenance). Second-order factors were considered but could not be tested with only two 2-item factors per scale. Model 2 included six correlated factors: two awareness factors, two maintenance factors, and the two initial adoption and implementation scales. Model fit improved, but remained poor (Table 2). In model 3, additional modifications were made to model 2 based on modification indices. We deleted two items with low factor loadings (<0.40) and one additional item due to the large number of modification indices suggesting correlated errors with other items. Two error covariances were added when model fit was improved and when inspection of the items revealed possible instrument or method effects (*e.g.*, items contained the same word or phrase). Fit indices for model 3 showed good fit (Table 2).

**Table 2:** Measurement model development for scales based on stages of organizational readiness (n = 243)

**Model**	**Model fit indices**					
	**χ**^**2**^	***df***	***p***	**CFI**	**RMSEA (90% CI)**	**AIC**
1. Initial four-factor model						
Awareness (4) + adoption (5) + implementation (5) + maintenance (9)	823.531	224	.000	.688	.105 (.098, .113)	973.531
2. Revised six-factor model						
Community awareness (2) + agency awareness (2) + adoption (5) + implementation (5) + resource maintenance (5) + evaluation maintenance (4)	578.494	215	.000	.811	.084 (.075, .092)	746.494
3. Revised six-factor model with additional modification						
Model 2 minus three items and added two error covariances	303.602	153	.000	.905	.064 (.053, .074)	457.602

CFI = comparative fit index; RMSEA = root mean square error of approximation; CI = confidence interval; AIC = Akaike information criteria.

All scale items and standardized factor loadings from model 3 are reported in Table 3. All items were significant indicators of their respective factor (*p* < .001). Intercorrelations between readiness scales in model 3 are shown in Table 4. Intercorrelations were generally highest between factors in adjacent stages, with the highest correlations between agency awareness and adoption (*r* = .83, *p* < .05) and between implementation and resource maintenance (*r* = .95, *p* < .05). Based on the high correlations, we explored the possible existence of a combined factor for these two sets of scales, but since model fit was worse they were retained as separate factors. The community awareness factor had the lowest correlations with other factors in the model.

**Table 3:** Standardized factor loadings from final structural equation model, with initial model factor loadings included for comparison

**Item**	Final model (Model 3, Table 2)^*^	**Initial model (Model 1, Table 2)**
Community awareness		
Community considers intervention a solution	.90	.24
Community considers [health issue] a problem	.58	.21
Agency awareness		
Agency leadership aware of sources for EBIs	.81	.78
Agency staff aware of sources for EBIs	.64	.66
Adoption		
Agency leadership encourages use of EBIs	.87	.83
EBIs are readily adopted in agency	.79	.78
Supervisor expects research evidence	.60	.68
Currently using research evidence^***^		.58
Access to help in utilizing research evidence^***^		.38
Implementation		
Agency has resource to implement intervention	.78	.77
Intervention has support of agency leadership	.74	.72
Agency adapts EBI to meet community needs	.66	.70
Intervention is supported by community leadership	.49	.50
Extent intervention team has necessary skills^**^	.49	.49
Resource maintenance		
Agency will continue to have intervention staff	.62	.59
Agency has diverse partners sharing resources	.50	.48
Agency has obtained range of funding sources	.44	.39
Agency has adequate fiscal policies	.43	.42
Intervention would continue if funding was lost^***^		.32
Evaluation maintenance		
Agency uses evaluation to monitor and improve	.85	.62
Agency had prior plan to evaluate intervention	.76	.58
Agency disseminates findings to community	.65	.58
Agency conducts community needs assessment	.48	.55

EBI = evidence-based intervention.

Note: error covariances included in the final model were between items 1 and 11 and between items 6 and 10.

^*^p values for all factor loadings were <.001.

^**^For this item, seven-point Likert scale ranged from 1 = not at all to 7 = completely (all other items: 1 = strongly disagree to 7 = strongly agree).

^***^Item deleted from final model.

**Table 4:** Intercorrelations among readiness scales

	**Agency awareness**	**Adoption**	**Implementation**	**Resource maintenance**	**Evaluation maintenance**
Community awareness	.23^*^	.12	.32^*^	.42^*^	.22^*^
Agency awareness		.83^*^	.60^*^	.39^*^	.30^*^
Adoption			.59^*^	.44^*^	.27^*^
Implementation				.95^*^	.49^*^
Resource maintenance					.53^*^

^*^p < .05.

### Performance of readiness scales

Internal reliability estimates based on Cronbach’s alpha are presented in Table 5 for the final, modified scales. Alphas were generally acceptable for all scales, except for the relatively low value for the resource maintenance scale. Test-retest reliability, as measured by the ICC, was found to be good to moderate across scales [37], ranging from 0.71 (adoption) to 0.47 (agency awareness).

**Table 5:** Characteristics of revised readiness scales and mean summary scores across program types (n = 243)

	**Scale characteristics**			Mean score (SD)^**^	**Mean score by program type**				
	**Items**	**α**	ICC^*^		**MPB grantee obesity programs**	**Non-MPB obesity programs**	**Tobacco programs**	**Diabetes programs**	**Asthma programs**
Final model									
Community awareness	2	.71	0.50	5.21 (1.13)	5.10	5.27	5.22	4.98	5.55
Agency awareness	2	.67	0.47	5.87 (0.96)	6.08	5.89	5.82	5.73	5.98
Adoption	3	.80	0.71	6.00 (0.99)	6.10	6.12	5.94	5.92	5.99
Implementation	5	.77	0.67	5.45 (0.91)	6.04	5.22^***^	5.48^***^	5.28^***^	5.26^***^
Evaluation maintenance	4	.75	0.67	5.73 (0.97)	5.77	5.27^***^	6.13	5.36	5.69
Resource maintenance	4	.57	0.68	4.67 (1.04)	5.46	4.56^***^	4.42^***^	4.77^***^	4.74^***^

SD = standard deviation; ICC = intraclass correlation; MPB = Model Practice Building.

^*^Subsample from test-retest study (n = 92).

^**^Response value ranged from 1 to 7.

^***^p < .05 for pairwise contrast with MPB grantee obesity programs as reference group in generalized linear model.

Mean scores derived from the revised scales of the final model were examined across program types (Table 5). The comparisons were interpreted based on *a priori* expectations for program status with respect to the readiness scale. As a result of prior work with the MPB grantee obesity program group (as described in Methods above), this group was expected to have higher readiness scores than most other groups, particularly for latter stages. Tobacco control programs were expected to have the next-highest level of readiness based on the longer history of established programs in this area. However, there were no differences across groups for awareness and adoption scores, with all groups scoring relatively high on all scales, with the exception of community awareness scales, which were slightly lower but not different across groups. The MPB grantee group had higher implementation scores than all other groups (*p* < .05).

An analysis of the association of the readiness factors with organizational climate and university affiliation provided an additional assessment of the performance of the scales in relation to possible mediators of readiness (Table 6). The significant positive correlations between organizational climate and most of the readiness scales (weaker correlations were observed for community awareness) indicate that an organization with a climate favorable to evidence-based practice is more likely to have higher readiness. Having a university affiliation was not associated with any of the readiness factors.

**Table 6:** Associations between readiness stages from the final measurement model with organizational climate and university affiliation (n = 243)

**Readiness stage**	**Organizational climate**	**University affiliation**
Community awareness	.22^*^	–.07
Agency awareness	.55^*^	–.06
Adoption	.62^*^	–.08
Implementation	.79^*^	–.08
Evaluation maintenance	.43^*^	.02
Resource maintenance	.71^*^	–.14

^*^p < .05.

## Discussion

This article describes the initial assessment of the measurement properties of a novel survey instrument to stage organizational readiness for EBIs in community-based chronic disease practice settings. We found support for our theory-based measures of readiness stages using CFA, though some modification was needed before arriving at a final model. Most notably, the awareness scale was split into two separate factors representing community and agency awareness, and the maintenance scale was split into two separate factors representing resource and evaluation maintenance. The mean scale scores followed a hypothesized pattern across types of programs, with the group of grant-funded programs that had received additional support exhibiting higher mean scores, particularly with respect to latter stages.

This staging survey is based on the underlying assumption that moving from one stage to another occurs successively, with strength in earlier stages serving as accumulated capacity to move to the next stage. Our analysis found some support for this, as correlations between adjacent stages were generally stronger than correlations between nonadjacent stages. However, this model may oversimplify the variation that exists in processes involved in moving from one stage to another. It may be that the organizational processes that lead to awareness and initial adoption not only differ from the processes that lead to implementation and maintenance but also that the actual system underlying the process is itself different. For example, it is possible that awareness is driven by a diffusion process, via linked networks of practitioners and organizations, but that implementation and maintenance are driven more by the multilevel systems and structure that drive capacity [38]. In our analysis, while the resource maintenance scale distinguished between programs according to *a priori* expectation of readiness, it was comprised of items with moderate to low factor loadings, which likely contributed to the moderate alpha for this final scale. It is possible that our scale didn’t fully capture the domains that comprise maintenance. Likewise, the awareness domain, which was split into subscales to achieve better model fit for the measurement instrument as a whole, resulted in two-item subscales, which is considered less than ideal for measurement of latent constructs. Future studies that can afford to increase the number of survey items may benefit from adding items designed to more comprehensively measure the different content domains within each stage.

We found that an organizational climate more favorable to using research evidence was related to all stages of readiness for evidence-based practice, with the caveat that our measure of implementation climate is new and its measurement properties are yet to be examined. This is in line with prior work that has suggested that having an organizational culture favorable to using evidence may moderate the effectiveness of strategies to increase the implementation of evidence-informed policies and programs in public health settings [39]. Organizational factors such as prioritizing use of evidence in practice have been found to predict adoption of evidence-based practice in state health departments [40]. This would suggest that in addition to staging readiness for evidence-based practice, assessing organizational context with respect to the culture and climate toward using evidence will be equally important in determining appropriate implementation strategies in a given setting. It may be that if organizational culture and climate toward using evidence is low, then strategies to enhance culture toward using evidence in general (*e.g.*, engaging leadership) may be considered as a possible starting point for implementation strategies.

This survey instrument may be grouped with other types of organizational assessments in the dissemination and implementation literature [41], most relevant being assessments of culture and climate [42] and readiness for change [43], although some distinctions are worth noting. Other organizational assessments include domains that do not have a clear sequenced relationship, while by definition, our framework’s stages are sequentially dependent on each other. While our assessment of readiness for EBIs in chronic disease prevention settings may be similar in orientation to instruments that measure readiness for change, our survey is more specific to organizational readiness for EBIs anchored in particular chronic disease program areas rather than the more global orientation of readiness for change. As Weiner [44] notes [43], the question of whether our instrument is measuring “readiness” or “determinants of readiness” may be important to consider when determining appropriate applications.

Another key point of departure for our instrument is that we were targeting public health chronic disease prevention settings versus clinical healthcare settings. The emphasis in public health settings is to deliver interventions using a community-based approach rather than an individual, patient-oriented approach, which is reflected in the structure and function of the organization, from program level on up [45]. These types of interventions tend to be carried out in teams within the organization, and often extend to community partners. Our instrument reflects this, as items comprising each stage include some assessment of agency staff, agency leadership, and the broader community (which could include individual community members as well as partners). Previous scales used to assess stages of EBI (*i.e.*, “innovation”) diffusion in public health organizations have been substantially longer than the one created for this project (*e.g.*, Steckler *et al.*[21], which had 125+ items).

This paper has a number of strengths and limitations worth noting. To our knowledge, our survey represents the first of its kind to be used in community and public health chronic disease prevention settings. The instrument is brief (less than six minutes average completion time in our sample) and easy to complete, as evidenced by the response rate, which suggests the feasibility of data collection for longitudinal assessments; our response rate was in line with previous work among local health officials (73% in Brownson *et al.*) [10]. The limited time and attention of chronic disease program staff to devote to a survey is no doubt shared across professional settings; this serves as a demonstration of the utility and validity of a brief instrument and may bolster efforts in other areas. We used a rigorous confirmatory analytic approach to confirm which items belonged to which content domains or scales. Additional testing is required to determine whether this measure of organizational readiness for EBIs is indeed predictive of actual adoption and implementation of EBIs. Our final model resulted in six scales, two of which (community and agency awareness) had only two items each, which is considered less than ideal for measurement of latent constructs. The final alphas for our scales were generally acceptable, especially considering the brevity of the scales, with the exception of the relatively low alpha for the resource maintenance scale. This suggests room for improvement in future versions of the instrument measuring these domains. We did not have sufficient sample size in this study to confirm our final model with a hold-out sample or to test for item invariance across multiple subgroups (*e.g.*, program type, urban vs. rural setting). It is possible that readiness for evidence-based practice may vary by type of practice setting and that different measures may be needed. In many cases there was one respondent representing an organization; it is yet unclear how many respondents it takes in a particular setting to precisely measure these issues and track changes over time. Future work could explore conceptual and analytical issues around level of measurement and clustering with multilevel data collection and analysis.

Another important consideration in interpreting and using this instrument is the basis by which evidence-based practice was assessed. For the current survey, we define evidence-based practice as “an evidence-based intervention, evidence-based policy, or evidence-based clinical care…that integrates science-based interventions with community preferences to improve the health of populations.” Examples of sources of evidence-based interventions provided to respondents in the body of the survey were based on systematic research reviews (*i.e.*, Community Guide, Cochrane). There may be additional information about community and organizational context, as well as knowledge derived from professional experience (practice-based evidence), that also comprise elements of evidence applied in practice settings [46] that were not explicitly addressed.

## Conclusions

The lack of adequate measurement tools hinders progress in dissemination and implementation research [47]. To help fill this void, these results describe the reliability and measurement properties of a theory-based tool; the short, user-friendly instrument may be useful to researchers and practitioners seeking to assess organizational readiness for evidenced-based practice across a variety of chronic disease prevention programs and settings. Although the field is young, it is likely that intervention strategies to enhance dissemination and implementation progress will need to be tailored to stage of readiness. This parallels the recognized need in clinical settings to generate more evidence to guide the choice of implementation strategies based on the expectation of relative effectiveness given the characteristics of a particular setting [48].

There are many potential applications of a survey instrument such as this, in addition to tailoring intervention strategies to stage of readiness. For example, the survey could be used to provide a gateway to initiating contact with practice settings and as a basis from which to seek additional, in-depth information using more qualitative approaches. In this light, this staging survey may be placed in the spectrum of participatory approaches [49], in which assessment of organizational readiness may serve as one part in the process of integrating knowledge about the practice setting by including input from practitioners into developing strategies to increase the use of evidence in that setting. There are numerous analytical applications of the quantitative measures of readiness stage, including examination of the relationship between stages and measures of program success and how this may differ across program areas. Finally, the scales derived from this survey instrument may also be considered as part of a spectrum of implementation outcomes [50] and could be explored as markers of success of an implementation strategy (*i.e.*, evaluate shift from low implementation to high implementation/maintenance).

## Competing interests

‘The authors declare that they have no competing interests’.

## Authors’ contributions

KAS carried out the CFA analysis, supervised all other data analysis, participated in survey development, and drafted the manuscript. AM supervised the CFA analysis and contributed to writing the manuscript. CF participated in data collection and management, conducted the test-retest reliability analysis, and contributed to writing the manuscript. EB participated in data collection and survey development and contributed to writing the manuscript. MD coordinated survey development, assisted with data collection and management, and contributed to writing the manuscript. DAL participated in the study design and survey development and contributed to writing the manuscript. RCB led the study design and survey development and contributed to writing the manuscript. All authors read and approved the final manuscript.

## Supplementary Material

Additional file 1 Prevention Program Assessment. Click here for file

## References

[B1] Xu J Kochanek KD Murphy SL Tejada-Vera B Deaths: final data for 2007 2010 National vital statistics reports, vol. 58 (19), Hyattsville, MD: National Center for Health Statistics 25075874

[B2] Anderson G Herbert R Zeffiro T Johnson N Chronic Conditions: Making the Case for Ongoing Care 2004 Partnership for Solutions, Johns Hopkins University, Baltimore

[B3] Flegal KM Graubard BI Williamson DF Gail MH Excess deaths associated with underweight, overweight, and obesity JAMA 2005 293 1861 1867 10.1001/jama.293.15.1861 15840860

[B4] Mokdad AH Marks JS Stroup DF Gerberding JL Actual causes of death in the United States, 2000 JAMA 2004 291 1238 1245 10.1001/jama.291.10.1238 15010446

[B5] Murray CJ Kulkarni SC Ezzati M Understanding the coronary heart disease versus total cardiovascular mortality paradox: a method to enhance the comparability of cardiovascular death statistics in the United States Circulation 2006 113 2071 2081 10.1161/CIRCULATIONAHA.105.595777 16636170

[B6] Cancer Control PLANET http://cancercontrolplanet.cancer.gov/index.html

[B7] Research-tested Interventions Programs http://rtips.cancer.gov/rtips/index.do

[B8] Guide to Community Preventive Services http://www.thecommunityguide.org/index.html

[B9] Guide to Clinical Preventive Services 2010-2011 http://www.ahrq.gov/clinic/pocketgd.htm

[B10] Brownson RC Ballew P Brown KL Elliott MB Haire-Joshu D Heath GW Kreuter MW The effect of disseminating evidence-based interventions that promote physical activity to health departments Am J Public Health 2007 97 1900 1907 10.2105/AJPH.2006.090399 17761575 PMC1994189

[B11] Zaza S, Briss PA, Harris KW The Guide to Community Preventive Services: What Works to Promote Health? 2005 Oxford University Press, New York

[B12] Brownson RC Fielding JE Maylahn CM Evidence-based public health: a fundamental concept for public health practice Annu Rev Public Health 2009 30 175 201 10.1146/annurev.publhealth.031308.100134 19296775

[B13] Dearing JW Evolution of diffusion and dissemination theory J Public Health Manag Pract 2008 14 99 108 18287914 10.1097/01.PHH.0000311886.98627.b7

[B14] Dearing JW Kee KF Brownson R, Colditz G, Proctor E Historical Roots of Dissemination and Implementation Science Dissemination and Implementation Research in Health: Translating Science to Practice 2012 Oxford University Press, Oxford in press

[B15] Gaglio B Glasgow RE Brownson R, Colditz G, Proctor E Evaluation Approaches for Dissemination and Implementation Research Dissemination and Implementation Research in Health: Translating Science to Practice 2012 Oxford University Press, Oxford in press

[B16] Rogers EM Diffusion of innovations 2003 Free Press, New York

[B17] Glasgow RE Vogt TM Boles SM Evaluating the public health impact of health promotion interventions: the RE-AIM framework Am J Public Health 1999 89 1322 1327 10.2105/AJPH.89.9.1322 10474547 PMC1508772

[B18] Balbach ED Using case studies to do program evaluation 1999 California Department of Health Services, Sacramento

[B19] Briss PA Brownson RC Fielding JE Zaza S Developing and using the Guide to Community Preventive Services: lessons learned about evidence-based public health Annu Rev Public Health 2004 25 281 302 10.1146/annurev.publhealth.25.050503.153933 15015921

[B20] Dreisinger ML Boland EM Filler CD Baker EA Hessel AS Brownson RC Contextual factors influencing readiness for dissemination of obesity prevention programs and policies Heal Educ Res 2012 27 292 306 10.1093/her/cyr063 21893684

[B21] Steckler A Goodman RM McLeroy KR Davis S Koch G Measuring the diffusion of innovative health promotion programs Am J Health Promot 1992 6 214 224 10.4278/0890-1171-6.3.214 10148679

[B22] Jacobs JA Dodson EA Baker EA Deshpande AD Brownson RC Barriers to evidence-based decision making in public health: a national survey of chronic disease practitioners Public Health Rep 2010 125 736 742 20873290 10.1177/003335491012500516 PMC2925010

[B23] Center for Tobacco Policy Research Program Sustainability Assessment Tool 2011 Center for Tobacco Policy Research, Brown School, Washington University in St. Louis, St. Louis http://ctpr.wustl.edu/documents/Sustainability_Tool_3.11.pdf

[B24] Jobe JB Mingay DJ Cognitive research improves questionnaires Am J Public Health 1989 79 1053 1055 10.2105/AJPH.79.8.1053 2751028 PMC1349911

[B25] Jobe JB Mingay DJ Cognitive laboratory approach to designing questionnaires for surveys of the elderly Public Health Rep 1990 105 518 524 2120731 PMC1580104

[B26] Streiner D Norman G Health Measurement Scales: A practical guide to their development and use 2006 3 Oxford University Press, New York

[B27] Willis GB Cognitive interviewing: a tool for improving questionnaire design 2005 Sage, Thousand Oaks

[B28] Luke DA Ribisl KM Walton MA Davidson WS Assessingthe diversity of personal beliefs about addiction: Development of the Addiction Belief Inventory Substance Use Misuse 2002 37 91 121 10.1081/ja-120001498 11848161

[B29] Maruyama GM Basics of structural equation modeling 1998 Sage Publications, Thousand Oaks, CA

[B30] Arbuckle JL Amos 16.0 User's Guide 2007 Amos Development Corporation, Chicago

[B31] Arbuckle JL Marcoulides GA Schumacker RE Full information estimation in the presence of incomplete data Advanced structural equation modeling: issues and techniques 1996 Erlbaum, Mahwah 243 277

[B32] Schafer JL Graham JW Missing data: Our view of the state of the art Psychol Meth 2002 7 147 177 12090408

[B33] Hu L Bentler PM Hoyle RH Evaluating model fit Structural equation modeling 1995 Sage Publications, Thousand Oaks 76 99

[B34] Hu L Bentler PM Cutoff criteria for fit indexes in covariance structure analysis: Conventional criteria versus new alternatives Struct Equ Model 1999 6 1 55 10.1080/10705519909540118

[B35] StataCorp Stata Statistical Software: Release 11 Book Stata Statistical Software: Release 11 2009 StataCorp LP, College Station, TX

[B36] Rousson V Gasser T Seifert B Assessing intrarater, interrater and test-retest reliability of continuous measurements Stat Med 2002 21 3431 3446 10.1002/sim.1253 12407682

[B37] Rosner B Fundamentals of Biostatistics 2010 7 Brooks/Cole, Boston

[B38] Greenhalgh T Robert G Macfarlane F Bate P Kyriakidou O Diffusion of innovations in service organizations: systematic review and recommendations Milbank Q 2004 82 581 629 10.1111/j.0887-378X.2004.00325.x 15595944 PMC2690184

[B39] Dobbins M Hanna SE Ciliska D Manske S Cameron R Mercer SL O'Mara L DeCorby K Robeson P A randomized controlled trial evaluating the impact of knowledge translation and exchange strategies Implement Sci 2009 4 61 10.1186/1748-5908-4-61 19775439 PMC2936828

[B40] Brownson RC Ballew P Dieffenderfer B Haire-Joshu D Heath GW Kreuter MW Myers BA Evidence-based interventions to promote physical activity: what contributes to dissemination by state health departments Am J Prev Med 2007 33 S66 S73 quiz S74-68 10.1016/j.amepre.2007.03.011 17584593

[B41] Aarons GA Horowitz JD Dlugosz LR Ehrhart MG Brownson RC, Colditz G, Proctor E The role of organizational processes in dissemination and implementation research Dissemination and Implementation Research in Health: Translating Science to Practice Oxford University Press, Oxford in press

[B42] Glisson C James LR The cross-level effects of organizational climate and culture in human service teams J Organ Behav 2002 23 767 794 10.1002/job.162

[B43] Weiner BJ Amick H Lee SY Conceptualization and measurement of organizational readiness for change: a review of the literature in health services research and other fields Med Care Res Rev 2008 65 379 436 10.1177/1077558708317802 18511812

[B44] Weiner BJ A theory of organizational readiness for change Implement Sci 2009 4 67 10.1186/1748-5908-4-67 19840381 PMC2770024

[B45] Stamatakis KA Vinson CA Kerner JF Brownson RC, Colditz GA, Proctor EK Dissemination and implementation research in community and public health settings Dissemination and Implementation Research in Health: Translating Science to Practice 2012 Oxford University Press, New York

[B46] Green LW Public health asks of systems science: to advance our evidence-based practice, can you help us get more practice-based evidence? Am J Public Health 2006 96 406 409 10.2105/AJPH.2005.066035 16449580 PMC1470512

[B47] Proctor E Brownson RC Brownson RC, Colditz G, Proctor E Measurement Issues in Dissemination and Implementation Research Dissemination and Implementation Research in Health: Translating Science to Practice Oxford University Press in press

[B48] Grimshaw J Eccles M Thomas R MacLennan G Ramsay C Fraser C Vale L Toward evidence-based quality improvement. Evidence (and its limitations) of the effectiveness of guideline dissemination and implementation strategies 1966-1998 J Gen Intern Med 2006 21 Suppl 2 S14 S20 16637955 10.1111/j.1525-1497.2006.00357.x PMC2557130

[B49] Cargo M Mercer SL The value and challenges of participatory research: strengthening its practice Annu Rev Public Health 2008 29 325 350 10.1146/annurev.publhealth.29.091307.083824 18173388

[B50] Proctor E Silmere H Raghavan R Hovmand P Aarons G Bunger A Griffey R Hensley M Outcomes for Implementation Research: Conceptual Distinctions, Measurement Challenges, and Research Agenda Adm Policy Ment Health 2010 38 65 76 20957426 10.1007/s10488-010-0319-7 PMC3068522

