# Supplementary material for: Measurement properties of a novel survey to assess stages of organizational readiness for evidence-based interventions in community chronic disease prevention settings
Source: Implement Sci. 2012 Jul 16;7:65. doi: 10.1186/1748-5908-7-65 (PMC3418158; doi:10.1186/1748-5908-7-65)
Supplement: Additional file 1 — Prevention Program Assessment. [file 1748-5908-7-65-S1.docx]

**Prevention Program Assessment**

This survey tool has been developed through collaboration between the Prevention Research Center in St. Louis and the Missouri Foundation for Health as a means of assisting 19 prevention interventions across the state of Missouri with dissemination.

**Definitions:**

To ensure we are on the same page with our terminology, we have provided you with a few definitions for terms that are used throughout our survey

The term **intervention** refers to a specific, structured intervention that could include behavioral strategies, changes to the physical environment, policy approaches, or clinical services. For the purpose of this survey, we are interested in the intervention that was referred to when recruiting you for participation.

We define an **evidence-based intervention, evidence based policy, or** **evidence based clinical care** as one that integrates science-based interventions with community preferences to improve the health of populations.

Some commonly recognized **sources for evidence-based** interventions such as:

- The Centers for Disease Control and Prevention’s Community Guide
- The reviews of the Cochrane Collaboration

We define **community** as those individuals who receive the intervention and/or benefit from the intervention. This could include groups such as local residents, organizational leaders, policy makers, or other stakeholders.

**Background Questions :**

1. **How do you best describe your agency/organization?**

- State Health Department
- Local Health Department
- Health care facility (e.g., hospital, clinic, health center)
- Coalition
- Collaborative
- Faith-based organization
- Advocacy Group
- University or School
- Community-Based Organization
- Voluntary Health Organization (e.g., American Cancer Society, American Heart Association, American Lung Association)
- Other government agencies
- Other (please specify)_________________________

1. **What is your role with the intervention (check all that apply)?**

- Principal investigator/Director
- Program developer
- Program manager/implementer
- Program evaluator
- Program staff
- Other (please specify)_________________________

1. **How long have you worked with the intervention?**

- Less than 5 years
- 5-10 years
- 11-15 years
- 16-19 years
- 20 or more years

1. **Check the one option that best describes your position within your agency or organization.**

- Program manager/administrator/coordinator
- Health educator
- Communications specialist
- Community organizer
- Epidemiologist
- Statistician
- Program planner
- Program evaluator
- Division or bureau head/Division Deputy Director
- Department head
- Nurse, social worker, dietician, nutritionist
- Academic researcher
- Academic educator
- Other (please specify)_________________________

1. **Describe the geographic region your organization serves (check all that apply):**

- Urban
- Rural
- Suburban

1. **Describe the geographic reach of your organization**

- Neighborhood
- Municipality
- County
- Multi-county
- City
- State
- Other (please specify)__________________________

1. **Does your organization have a *formal affiliation* with a university? (this includes official relationships to collaborate on research, education and service)**

- Yes
- No

1. **How many staff (full and part-time) members are currently working on the intervention?**

**COMMUNITY AWARENESS**

This section of the survey will help us to understand your target population’s awareness of the disease burden. For the following statements, please indicate the extent to which you agree or disagree.

1. **The community served by the intervention considers obesity to be a problem.**

Strongly Disagree Strongly Agree

1. 2 3 4 5 6 7
2. **The community served by the intervention considers it to be a solution to the problem.**

Strongly Disagree Strongly Agree

1 2 3 4 5 6 7

**AGENCY AWARENESS:**

We would also like to learn more about your agency’s awareness of sources for evidence-based interventions. For the following statements, please indicate the extent to which you agree or disagree.

1. **To what extent is the leadership within your agency aware of sources for evidence-based interventions?**

Not at all Completely

1 2 3 4 5 6 7

1. **To what extent is the intervention staff within your agency aware of sources for evidence-based interventions?**

Not at all Completely

1 2 3 4 5 6 7

**ADOPTION:**

This next section of the survey will help us to understand your agency’s adoption of evidence-based strategies and resources. For the following statements, please indicate the extent to which you agree or disagree.

1. **Leadership within your agency encourages the use of evidence-based** **interventions to guide the intervention efforts.**

Strongly Disagree Strongly Agree

1 2 3 4 5 6 7

1. **Evidence-based interventions are readily adopted within your agency.**

Strongly Disagree Strongly Agree

1 2 3 4 5 6 7

1. **My direct supervisor expects me to include research evidence in decision making related to planning the intervention.**

Strongly Disagree Strongly Agree

1 2 3 4 5 6 7

**IMPLEMENTATION:**

This next section of the survey will help us understand your agency’s intervention implementation. For the following statements, please indicate the extent to which you agree or disagree.

1. **When an evidence-based intervention is available, your agency is able to adapt it to meet the needs of the community it serves.**

Strongly Disagree Strongly Agree

1 2 3 4 5 6 7

1. **Your agency currently has the resources (staff, facilities, partners etc.) to implement the intervention.**

Strongly Disagree Strongly Agree

1 2 3 4 5 6 7

1. **The intervention has support from the leadership within your agency.**

Strongly Disagree Strongly Agree

1 2 3 4 5 6 7

1. **The intervention is supported by the leadership within the community (for example: a mayor, city council, school board).**

Strongly Disagree Strongly Agree

1 2 3 4 5 6 7

1. **To what extent does the team carrying out the intervention have the necessary skills?**

Not at all Completely

1 2 3 4 5 6 7

**RESOURCE MAINTENANCE:**

This next section of the survey helps us to understand resources and procedures that may be important for the maintenance or sustainability of the intervention. For the following statements, please indicate the extent to which you agree or disagree.

1. **Your agency engages a diverse network of partners who actively support the intervention through the sharing of resources and/or participation in planning activities.**

Strongly Disagree Strongly Agree

1 2 3 4 5 6 7

1. **Your agency will continue to have the staff needed to implement the intervention.**

Strongly Disagree Strongly Agree

1 2 3 4 5 6 7

1. **Your agency has adequate fiscal policies and procedures in place to ensure proper use of the intervention funding.**

Strongly Disagree Strongly Agree

1 2 3 4 5 6 7

1. **Your agency has obtained a range of funding sources for the intervention so you are less dependent on one funder.**

Strongly Disagree Strongly Agree

1 2 3 4 5 6 7

**EVALUATION MAINTENANCE:**

This next section of the survey helps us to understand evaluation and dissemination actvities that may be important for the maintenance or sustainability of the intervention. For the following statements, please indicate the extent to which you agree or disagree.

1. **Your agency conducts periodic needs assessments of the community to make sure that the intervention continues to meet their needs.**

Strongly Disagree Strongly Agree

1 2 3 4 5 6 7

1. **Your agency planned for evaluation of the intervention prior to implementation.**

Strongly Disagree Strongly Agree

1 2 3 4 5 6 7

1. **Your agency uses evaluation data to monitor and improve the intervention.**

Strongly Disagree Strongly Agree

1 2 3 4 5 6 7

1. **Your agency disseminates evaluation findings from the intervention to important community groups.**

Strongly Disagree Strongly Agree

1 2 3 4 5 6 7

**ORGANIZATIONAL CLIMATE:**

This next section of this survey helps us to understand the organizational climate within your agency. For the following statements, please indicate the extent to which you agree or disagree.

1. **When decisions are being made within your agency, the intervention staff affected are asked for their input.**

Strongly Disagree Strongly Agree

1 2 3 4 5 6 7

1. **To what extent is your agency willing to make changes (e.g., enhance workforce training, seek out new partners) in order to enable the use of evidence-based interventions?**

Not at all Completely

1 2 3 4 5 6 7

1. **To what extent is information widely shared in your agency so that everyone who makes decisions has access to all available knowledge?**

Not at all Completely

1 2 3 4 5 6 7

**If you have any thoughts or comments on the survey, please let us know your feedback: ­­­­­­­­­­­­____________________________________________________________________**

**Thank you for taking the time to complete our survey!**

­___________________________________________________________________________________
